# Quality of care provided in two Scottish rural community maternity units: a retrospective case review

**DOI:** 10.1186/s12884-017-1374-9

**Published:** 2017-06-21

**Authors:** Sara Denham, Tracy Humphrey, Ruth Taylor

**Affiliations:** 1 Robert Gordon University, Garthdee Road, Aberdeen, AB10 7QG UK; 2 Edinburgh Napier University, Sighthill Campus, Edinburgh, EH11 4BN UK; 3 Anglia Ruskin University, East Road Campus, Cambridge, CB1 1PT UK

**Keywords:** Rural maternity care, Safe maternity care, Effective maternity care, Midwife-led care, Maternity collaboration

## Abstract

**Background:**

Women in Scotland with uncomplicated pregnancies are encouraged by professional bodies and national guidelines to access community based models of midwife-led care for their labour and birth. The evidence base for these guidelines relates to comparisons of predominantly urban birth settings in England. There appears to be little evidence available about the quality of the care during the antenatal, birth and post birth periods available for women within the Scottish Community Maternity Unit (CMU) model.

The research aim was to explore the safety and effectiveness of the maternity services provided at two rural Community Maternity Units in Scotland, both 40 miles by main road access from a tertiary obstetric unit.

**Methods:**

Following appropriate NHS and University ethical approval, an anonymous retrospective review of consecutive maternity records for all women who accessed care at the CMUs over a 12 month period (June 2011 to May 2012) was undertaken in 2013 -14. Data was extracted using variables chosen to provide a description of the socio-demographics of the cohort and the process and outcomes of the care provided. Data were analysed using descriptive statistics.

**Results:**

Regarding effectiveness, the correct care pathway was allocated to 97.5% of women, early access to antenatal care achieved by 95.7% of women, 94.8% of women at one CMU received continuity of carer and 78.6% of those clinically eligible accessed care in labour. 11.9% were appropriately transferred to obstetrician-led care antenatally and 16.9% were transferred in labour. All women received one-to one care in labour and 67.1% of babies born at the CMUs were breastfed at birth.

Regarding safety, severe morbidity for women was rare, perineal trauma of 3rd degree tear occurred for 0.3% of women and 1.0% experienced an episiotomy. Severe post partum haemorrhage occurred for 0.3% of women. Babies admitted to the Neonatal unit were discharged within 48 hrs.

**Conclusion:**

These findings support the recommendations of professional bodies and national guidelines. Maternity service provision at rural CMUs achieved a consistently high standard of safety and effectiveness when measured against national standards and international evidence.

## Background

Current expert guidance from professional bodies and national guidelines [1–3] encourage women with no morbidities and uncomplicated pregnancies to access community based models of midwife-led care. The guidance is based on primary evidence about safety that relates predominantly to births in urban settings in England. There appears to be little evidence available about the quality of the care during the entire childbirth continuum within the Scottish rural context [4].

In Scotland, Community Maternity Units (CMUs) were defined by the Expert Group on Acute Maternity Services in 2002 [5] as “*midwife-led, stand alone maternity units appropriately equipped for normal care with agreed transfer guidelines to a linked maternity unit.*” However, the definition fails to recognise that CMUs also provide care during pregnancy and the postnatal period for most women in the local community. Fewer women access care during labour and birth in these settings [6, 7].

Policy stipulates that the choice of care venue and lead professional should remain with women, however clinically appropriate maternity care pathways [8] are recommended to each woman depending on her individual needs. Since their implementation in 2009, no evidence has been published about the impact on quality of care for women and families.

### Current policy context

Table 1 shows a summary of policy and empirical evidence which contribute to show the currently available benchmarks of maternity care.

**Table 1:** Benchmarks of safe and effective maternity care

Year	Publication	Standard
2009	KCND Maternity Care Pathways [8]	Multidisciplinary maternity risk assessment and care pathways Antenatal: Midwife first point of contact clinician who conducts initial risk assessment recommends clinically appropriate care pathway 9 antenatal visits in first and 7 antenatal visits in second and subsequent pregnancy up to 40 weeks. Labour: Continuous assessment, appropriate birth environment and reduction of inappropriate interventions. Postnatal: Continuity of carer and person centred care plans.
2010	NHS Scotland Healthcare Quality Strategy [11]	Healthcare Quality Ambitions. Safe: no avoidable injury or harm to people from the healthcare they receive. Effective: appropriate treatments; interventions; support and services will be provided at the right time to those who will benefit and harmful variation will be eradicated.
2011	Birthplace Study [44]	National evidence of 21.9% transfer rate in labour from freestanding CMU to Obstetric Unit.
2011	The Refreshed Framework for Maternity Care [12]	Ten Principles of Maternity Care, but no specific benchmarks offered. Key Quality Indicators: Assets based and outcomes focussed maternity services aiming to improve the equity and quality of maternity care
2014	Health Efficiency and Access Target [13]	Antenatal Access: *“At least 80% of pregnant women in each SIMD quintile will have booked for antenatal care by the 12th week of gestation by March 2015.*
2016	NICE [3]	Antenatal guidance: Women are supported to access antenatal care, ideally by 10 weeks. Antenatal care should take place in a location that women can easily access. Intrapartum guidance: Women in established labour should have one-to - one care and support from an assigned midwife.
2015	Healthcare Improvement Scotland: Maternity and Children Quality Improvement Collaborative [15]	Reduce the incidence of avoidable harm in women and babies by 30% by 2015.
2016	Better Births [16]	Continuity of carer: “to ensure safe care based on a relationship of mutual trust and respect in line with the woman’s decisions. Every woman should have a midwife, who is part of a small team of 4 to 6 midwives, based in the community who knows the women and family, and can provide continuity throughout the pregnancy, birth and postnatally”. Safer Care: “professionals working together across boundaries to ensure rapid referral and access to the right care in the right place”.

The Keeping Childbirth Natural and Dynamic programme developed Maternity Care Pathways [8] in collaboration with the muliti-disciplinary maternity care team. The pathways were firmly based on evidence to inform and standardise a robust, dynamic and holistic approach to risk assessment throughout a woman’s maternity journey [9]. These maternity care pathways provide specific guidance which helped provide clear benchmarking for the provision of safe and effective maternity care against a background of policy aiming to reduce healthcare inequalities.

One of the key driving forces for maternity policy has been the reduction of inequalities in maternal and infant outcomes at birth, which have consistently been the focus of UK Government initiatives for the past decade during a period of financial and workforce challenges [2, 10].

NHS Scotland’s Healthcare Quality Strategy [11] (HQS) aimed to maximise the contribution of NHS Scotland to the creation of sustainable economic growth by improving health. It again focussed on reducing inequalities across the Scottish population. The HQS [11] was based on consultation with the people of Scotland, but a lack of established theoretical underpinnings became apparent when it was launched, as it was lacking valid measurement tools.

Building on the founding principles of safe, effective and person centred care used in the HQS [11], the Refreshed Framework for Maternity Care in Scotland: The Maternity Services Action Group was published [12]. This attempted to provide an overarching structure to facilitate the planning and provision of high quality and outcome focussed maternity services. The ambition to reduce inequalities in maternal and infant outcomes at birth was clearly stated, however the framework remained advisory and devoid of a clear implementation structure.

Early access to antenatal care is a key Scottish Government Health improvement, Efficiency and governance, Access to services and Treatment appropriate (HEAT) target [13]. This target aimed to clearly show whether implementation of a strategy to reduce inequalities in access to maternity care had been achieved. The target (shown in Table 1) was one of the strategic priority areas in NHS Board performance standards for local delivery plans for 2015 – 2016. By March 2016 the standard had been exceeded and in the poorest performing Scottish Index of Multiple Deprivation, 85.9% of women had booked for antenatal care by 12 weeks [14].

Despite the unique example of the HEAT target, the lack of an appropriate framework to assess the quality of care provision has been recognised. Consultation has recently begun by Healthcare Improvement Scotland [15] towards a comprehensive approach to reviewing the quality of care provided. Healthcare Improvement Scotland has introduced the Maternity and Children Quality Improvement Collaborative, which encompasses the activity of the Scottish Patient Safety Programme through its maternity care strand, with an overall aim which reflects that of the Refreshed Framework, to:

"*Improve outcomes and reduce inequalities in outcomes by providing a safe, high quality care experience for all women, babies and families across maternity care settings in Scotland*" [15].

The Better Births report of the National Maternity Review in England [16] was conducted against a background of failings in the quality of maternity care highlighted by the report of the Morecambe Bay Investigation, which suggested that a framework for safe, sustainable rural maternity services was developed. Better Births placed continuity of carer as the key to the delivery of increased safety, more personalised care, better support for vulnerable women with improved postnatal and perinatal mental health services.

Women in the UK are offered a choice of four birth locations: home; freestanding maternity unit (a CMU); midwife-led birthing unit alongside an obstetrician-led unit (OU) or within the labour ward of an obstetric unit. The availability to each woman of all four options depends on local service provision and their individual clinical circumstances. Their healthcare practitioner makes recommendations on the appropriateness of the locations for each woman depending on the risk of complications to mother and baby during labour and birth.

Women view safety against a background of accessible medical services in an affluent country [17]. They expect both their own and their babies’ survival both physically and emotionally to becoming confident mothers by minimising emotional harm [18]. Research exploring the factors influencing women’s choices about where to give birth, for example [17, 19–21], has predominantly found that the strongest influence is that of women’s concepts of safety. Some women who saw birth in terms of a risk laden process, chose birth in an Obstetric Unit as a method of mitigating their own risk and increasing the safety for their baby [22]. Those who chose to give birth in a midwife-led unit saw safety in terms of proximity to home and the ability to maintain control over their environment with the option to transfer to an Obstetric Unit should complications arise. The Birthplace in England Collaborative Group study collected data from over 68,000 women and confirmed that birth in a freestanding midwife-led unit was as safe and more cost effective than birth in an Obstetric Unit or a midwife-led unit attached to an Obstetric Unit [6, 23].

Studies of rural midwife-led maternity care provision outside the United Kingdom, mainly in Denmark [24], Finland [25], Sweden [26] and Australia [27] have also concluded that midwife-led maternity care during labour and birth is at least as safe as care in an Obstetric Unit. Ireland et al. [28] warn that accurate risk assessment and timely action are particularly important in rural midwife-led settings and recommend the use of local evidence based guidelines to make positive changes in the process and outcomes of rural maternity care [29].

The literature on maternity care provision regarding safe care, suggests that the safety of women and babies in terms of no avoidable injury occurring, depends not only on the outcomes of that care, but also the appropriateness of the environment provided for the delivery of healthcare services. Evidence presented in this paper regarding the rural CMU model of maternity care provision is not only important in order to assess and evaluate the safety and effectiveness of the care provided, but also to inform the sustainability of accessible local maternity services against a background of less than 3% of Scotland’s annual births occurring at CMUs [30].

The aim of this article is to report the findings from one phase of a case study using mixed methods which investigated the quality of care provided at two rural CMUs in Scotland. This paper presents an exploration of the safety and effectiveness of the maternity services provided at two rural CMUs through descriptive quantitative data collected through a case note review. To achieve this the socio-demographic and clinical characteristics of women who accessed care at the CMUs are described. The processes of care and clinical outcomes are also described and then compared with national pathways and guidance.

## Methods

### Setting and selection of CMUs

National data [30] were explored to identify rural midwife-led CMUs which had clinical activity of over 150 births annually, were situated at least 30 miles (or 30 min transfer time) from an OU [31] and had no on site obstetrician support. Those with under 150 births annually, those which reported a caesarean section rate and those situated within 30 min transfer time to an OU were excluded. Two rural CMUs from different Health Board areas fulfilled this criteria and the Heads of Midwifery were approached to ascertain their support for the CMUs to take part. Permission was obtained for both CMUs to be included in the study. These CMUs were situated within community hospitals in the heart of small towns, with main road access to the nearest tertiary referral obstetric units both approximately 40 miles away.

### Ethical considerations

The study received ethical approval from the local NHS Research Ethics Services. Appropriate management permission for site access was also obtained from gatekeepers (Heads of Midwifery and local records managers).

Anonymity and confidentiality of the CMUs were achieved by using the pseudonyms Seaview and Cherrytrees and by removing identifiable data during collection and dissemination of the study findings.

### Data collection

Anonymous retrospective data were extracted during 2013 - 2014 from a consecutive series of case notes (maternity records) of all women who accessed any maternity care during their maternity journey at the CMUs during a previous 12 month period (1st June 2011 to 31st May 2012). Standards for record keeping [3, 32] state that care provided should be recorded in clinical records and this is a requirement of both employers and regulators. Case note reviews have been previously used [8, 22] to determine both clinical appropriateness of care and describe outcome indicators of maternity care models in rural Scotland. No power calculation had been included in these studies. Significant complications of pregnancy, labour and the post-birth period are uncommon and the Birthplace study authors estimated that 28,000 planned midwifery unit births were required to detect differences in the safety of care provided in a comparative study of maternal perinatal and fetal birth outcomes. In 2011, there were just under 1800 births in all Scottish community maternity units, including those in urban locations and in island locations with obstetrician and general practitioner input. A review of all case notes of women who accessed care at the two rural CMUs, where just under 400 births had occurred in the previous 12- months, over a 12 month period was considered appropriate to assess the management of risk and adverse events to provide a descriptive overview of clinical rather than statistical significance of the care delivered at the CMUs.

### Selection of variables

The variables collected were selected from those used in Delivered with Care, a national survey of women’s experience of maternity care [33]. Redshaw and Heikila’s 2010 study aimed to provide a benchmark of current practice of care provision and a baseline for measuring change in the future. It also enabled comparison between care in different settings and units and covered outcomes of maternity care throughout the pregnancy episode. Variables were identified to address the specific objectives of the study. These included socio-demographic variables such as: age; ethnicity and pregnancy model of care. Processes of care and clinical outcomes were described by variables such as: the correct allocation of lead professional; planned place of birth; transfers to obstetrician-led care; type of birth; blood loss and resuscitation requirements of the babies. Full details of the variables are shown in the results section.

The local clinical guidelines were collected from each CMU. These guidelines were based on robust, evidence based national pathways. The hierarchy of evidence would place national guidelines above local clinical guidelines, as would the Nursing and Midwifery Council regulatory body [32] who expect midwives to practice based on evidence. To allow for standardised comparisons with other units, nationally recognised pathways of maternity care [8] and definitions of interventions [34] were used to assess the appropriateness of the care recorded in the case notes as delivered by the midwives to the women.

Any identifiable data collected from maternity records were anonymised by collapsing or categorising, for example categorising postcodes into their appropriate Scottish Index of Multiple Deprivation category. Data validation was achieved by using filters on the spreadsheet to identify internal consistency and reveal data entry errors of data clusters, unusual or implausible entries and missing data allowing frequency checks. Cross tabs (pivot tables) were also used at the end of every data collection episode. If inaccuracies were found, the records were re-checked and data entries corrected.

### Data storage

The anonymised data were securely stored and password protected in accordance with the university policy and practise, and the Data Protection Act 1998.

### Data analysis

Descriptive statistical analysis was undertaken using Statistical Package for the Social Sciences (SPSS), version 21, a statistical analysis software package.

## Results

In total, the rural CMU teams provided antenatal (including pre-pregnancy counselling, ultrasound scanning, parent education and co-ordinating obstetrician-led clinics) and postnatal care to 683 women. As shown in Table 2, the majority of women who accessed maternity care at both CMUs were British, aged between 26 and 30 years and were employed. Most women were living in the Scottish Index of Multiple Deprivation datazone allocated quintile of deprivation areas 3 and 4, (1 is considered to be the most deprived and 5 the least deprived).

**Table 2:** Socio-demographic and clinical characteristics of the women who accessed maternity care at the CMUs

CMU	Seaview		Cherrytrees		Total	
Characteristic	Frequency	Percentage (%)	Frequency	Percentage (%)	Frequency	Percentage (%)
Maternal age (years)						
15-20	41	10.8	29	9.6	70	10.2
21-25	111	29.0	79	26.2	190	27.8
26-30	124	32.5	97	32.1	221	32.3
31-35	71	18.6	77	25.5	148	21.6
36 and over	34	9.1	20	6.6	54	8.0
Total	381	100	302	100	683	100
Nationality						
White British	337	88.5	274	90.7	611	89.5
East European	37	9.7	10	3.3	47	6.9
Asian & African	4	1.0	9	3.0	13	1.9
Other European	3	0.8	9	3.0	12	1.7
Total	381	100	302	100	683	100
Previous Births						
None	177	46.4	145	48.0	322	47.2
One	131	34.4	98	32.5	229	33.5
Two or more	73	19.2	59	19.5	132	19.3
Total	381	100	302	100	683	100
Scottish Index of Multiple Deprivation						
Quintile 1	34	8.9	16	5.3	50	7.3
Quintile 2	77	20.2	89	29.5	166	24.3
Quintile 3	111	29.2	82	27.3	193	28.2
Quintile 4	114	29.9	91	30.1	205	30.1
Quintile 5	45	11.8	24	7.8	69	10.1
Total	381	100	302	100	681	100

The effectiveness of the processes of care and safety of the outcomes of the care provided at the CMUs are presented in the following two sections: effectiveness and safety.

### Effectiveness

The clinical characteristics of the women recorded by their midwives in their case notes were used to assess the pregnancy model of care allocated at booking. This determined the clinical care pathway recommended for women. Almost three quarters of the women ( *n* = 486, 71.2%) were allocated a midwife-led model of care at booking as they had no significant morbidity or obstetric risk factors. Appropriate assessment and referral through the initial risk assessment at booking occurred for 97.5% ( *n* = 666) of women where a midwife-led, obstetrician-led or referral for assessment by the maternity care team care pathway was recorded in the case notes at the initial assessment. The most common error was that the lead carer in pregnancy had been incorrectly recorded in the case notes (pregnancy records) reviewed. The records, however, in the antenatal visits section, revealed that the women actually had appropriate referrals made and were allocated correct care pathways. Antenatal transfers of care pathways from midwife-led to obstetrician-led occurred appropriately for 11.9% *n* = 58 women, the most common reason for transfer given by the midwives in the case notes was for post maturity.

As shown in Table 3, the majority of women (*n*= 654, 95.7%) accessed maternity care (usually referred to as a booking appointment) within the first 12 weeks of pregnancy, but 29 (4.3%) women accessed maternity care later. Women were found to be less likely to access early antenatal care when expecting a second or subsequent baby, they were following a midwife-led care pathway and were living in SIMD quintiles three and four.

**Table 3:** Clinician, gestation and allocated care pathways at booking

CMU	Seaview		Cherrytrees		Total	
Characteristic	Frequency	Percentage (%)	Frequency	Percentage (%)	Frequency	Percentage (%)
1st Point of Contact Clinician						
Midwife	372	97.6	268	88.7	640	93.7
GP/Other	9	2.4	34	11.3	43	6.3
Total	381	100	302	100	683	100
Gestation						
Before 12 weeks	363	95.3	291	96.4	654	95.7
After 12 weeks	18	4.7	11	3.6	29	4.3
Total	381	100	302	100	683	100
Care Pathway						
Midwife-led	259	68.0	227	75.1	486	71.2
Obstetrician-led	122	32.0	75	24.9	197	28.8
Total	381	100	302	100	683	100

The first healthcare professional the women contacted to access maternity care was a midwife for 640 (93.7%) women. Differences between the units may have occurred as local GP practices in Seaview’s catchment area referred women directly to the CMU for all maternity visits when they had confirmed their own pregnancies. In the Cherrytrees area some GPs continued to see women for an initial visit before referring them to their local CMU.

Continuity of carer, defined during a recent survey [35] as seeing the same midwife all or most of the time during pregnancy varied widely between the two CMUs. Antenatally 361 (94.8%) women at Seaview saw 3 midwives or fewer, but this was achieved for only 95 (31.3%) women at Cherrytrees where a team rather than named midwife approach to antenatal care was used.

Of the 486 women who were allocated a midwife-led care pathway at booking, 446 (91.7%) expressed a preference to give birth at their CMU in late pregnancy. By the onset of labour, 382 (78.6%) of these women accessed care at their CMU and 325 (66.9% of those who were allocated midwife-led care and 47.6% of all women who accessed care at the CMUs at booking) gave birth at the rural CMUs. Table 4 shows the birthplace preferences of all women who accessed care at the CMUs at three key stages in their pregnancies.

**Table 4:** Birthplace preferences of all women who accessed care at the CMUs

CMU	Seaview		Cherrytrees		Total	
Intended Birthplace	Frequency	Percentage (%)	Frequency	Percentage (%)	Frequency	Percentage (%)
At Booking						
CMU	197	51.6	199	65.9	396	58.0
Obstetric Unit	130	34.0	56	18.5	186	27.2
Undecided	42	11.0	42	13.9	84	12.3
Other Midwife-led Unit	9	2.5	2	0.7	11	1.6
Home	3	0.9	3	1.0	6	0.9
Total	381	100	302	100	683	100
At 36 weeks						
CMU	221	58.0	225	74.5	446	65.3
Obstetric Unit	127	33.3	66	21.9	193	28.3
Undecided	8	2.1	1	0.3	9	1.3
Other Midwife-led Unit	16	4.2	5	1.6	21	3.1
Home	1	0.3	2	0.7	3	0.4
Given Birth	8	2.1	3	1.0	11	1.6
Total	381	100	302	100	683	100
At Onset of Labour						
CMU	194	50.9	188	62.2	382	56.0
Obstetric Unit	154	40.4	105	34.8	259	37.9
Other Midwife-led Unit	26	6.8	2	0.7	28	4.1
Home	0	0.0	4	1.3	4	0.6
Given Birth	7	1.9	3	1.0	10	1.4
Total	381	100	302	100	683	100

The number of women who intended to give birth at the CMUs, rose by 7.3% (*n* = 50) between booking and 36 weeks at both CMUs, as women experienced the care and facilities available during their antenatal journeys. Attendance at antenatal classes was not recorded in the case notes, so this influence on decision making could not be explored in this study. An increase in the number of women accessing care in labour at the Obstetric Unit can be attributed to 58 (11.9%) women developing complications of pregnancy. The choice of giving birth at the CMUs could not be differentiated by clinical or socio-demographic characteristics. Of the women who planned to give birth at their CMU at 36 weeks, just over half, 51.1% (*n* = 113) at Seaview and 51.5% (*n*= 119) at Cherrytrees, were primiparous. Women who followed a midwife led care pathway but planned to give birth at an obstetrician-led unit (*n* = 30 at Seaview and *n *= 12 at Cherrytrees) were predominantly expecting their first baby (73.3%, *n* = 22 at Seaview and 83.3%, *n* = 10 at Cherrytrees). No other differences were observed in the women’s age, SIMD quintiles or nationalities between those who planned to give birth at CMU or OU settings.

Appropriate and timely transfer in labour was assessed against the NHS Quality Improvement Scotland [7] guidance and occurred for 16.9%, *n* = 66 women. The most common reason recorded in the case notes by the midwives for the transfer was given as delayed progress in the first stage of labour.

The most frequently used methods of pain management during labour differed between the two CMUs. Inhaled Entonox was used by 112 (63.8%) of the 164 women who gave birth at Seaview, and 29 (17.7%) chose to use intramuscular morphine. No birthing pools were available for use at Seaview. Water immersion was recorded for pain management for 142 (90.4%) of the 161 women who gave birth with the Cherrytrees team, 72% of which were waterbirths, and 12 (7.5%) women used morphine. Morphine is an opiate with the ability to cross the placenta and depress the respiratory drive of the baby at birth [36] and the use of morphine during labour has an impact on the condition of neonates at birth. As shown in Table 5, six (3.6%) babies required basic resuscitation assistance at birth to establish spontaneous regular respiration at Seaview, compared to two (1.2%) at Cherrytrees. No babies required admission at birth to the neonatal unit at the Obstetric Unit.

**Table 5:** Resuscitation requirements of babies born at the CMUs

CMU	Seaview		Cherrytrees		Total	
Resuscitation Performed	Frequency	Percentage (%)	Frequency	Percentage (%)	Frequency	Percentage (%)
None	149	90.8	156	96.9	305	93.9
Stimulation	9	5.5	3	1.9	12	3.7
Bag and Mask ventilation	6	3.7	2	1.2	8	2.4
Intubation	0	0.0	0	0.0	0	0.0
Total	164	100	161	100	325	100

All women received one-to - one care from a midwife during labour.

Two thirds of the babies (*n* = 218, 67.1%) born at the CMUs were breast fed at birth, and over half (*n* = 192, 59.1%) continued to be breast fed on transfer home. Over half (*n*= 352, 51.5%) of all women who accessed care at the CMUs (*n*= 683), were breastfeeding their babies on transfer from the care of the CMU teams to their health visitor.

### Safety

Interventions in labour, assessed against the Royal College of Midwives [34] definition of normal labour, were uncommon. Seven women (1.8%) who accessed care in labour across both CMUs experienced an artificial rupture of membranes, which were recorded by the midwives in the case notes to have been carried out to accelerate delayed progress in labour before considering transfer to an Obstetric Unit.

Three women (1.8%) having their first baby at Seaview experienced an episiotomy. They had spontaneous vaginal (land as opposed to water) births and no evidence of fetal compromise was documented in their records. This would question the appropriateness of this intervention. Evidence suggests that episiotomies should only be used when fetal compromise is suspected or during an instrumental delivery [7]. The degree of perineal trauma recorded by the midwives in women’s case notes differed between the CMUs, as shown in Table 6, and the availability of waterbirths at Cherrytrees may have had an influence on this. Just over one third (*n* = 42) of the 115 women experiencing a waterbirth were having their first baby, and just over half (52.4%, *n*= 22) of these women experienced no perineal trauma. Only 22.8% (*n* = 5) of the 22 women experiencing a non-waterbirth of their first baby at Cherrytrees sustained no perineal trauma.

**Table 6:** Perineal Trauma Sustained During Births at the CMUs

CMU	Seaview		Cherrytrees		Total	
Degree of Perineal Trauma	Frequency	Percentage (%)	Frequency	Percentage (%)	Frequency	Percentage (%)
None	70	42.7	93	57.8	163	50.1
First Degree	53	32.3	50	31.1	103	31.7
Second Degree	37	22.6	18	11.1	55	16.9
Third Degree	1	0.6	0	0.0	1	0.3
Episiotomy	3	1.8	0	0.0	3	1.0
Total	164	100	161	100	325	100

Two women experienced an appropriately managed, by assessing the record of recognition and subsequent actions by the midwives in the case records, against national care pathways [7], post partum haemorrhage of over 1,000mls.

All 325 women who gave birth at the CMUs, experienced spontaneous vaginal births. Of those who were transferred in labour (*n*= 66) to the Obstetric Unit, 33 (50.0%) went on to have a spontaneous vaginal birth, 16 (24.3%) had an assisted vaginal birth and 17 (25.7%) gave birth by caesarean section.

The babies born at the CMUs had a mean birthweight of 3.480 kgs. The resuscitation requirements of these babies are shown in Table 5. Three babies (1.8%) born at Seaview were admitted to the neonatal unit at the Obstetric Unit referral centre. All were over 12 hours old at transfer and were discharged within 48 h. Discharge from a neonatal unit within 48 h is a proxy measure for where there was no significant morbidity for the baby, as the short stay is most likely to be for assessment of the initial reason for admission [22].

There were no maternal or neonatal admissions to intensive care units.

## Discussion

This records review shows that maternity care provided at the two CMUs was delivered safely and effectively for most women. Three women (1% of those who gave birth at the CMUs) received less effective care as they experienced the intervention of an episiotomy in labour without a clearly documented reason.

At the first point of care, midwives at the CMUs accurately assessed women’s clinical needs and 97.5% were allocated maternity care pathways with the appropriate clinically recommended lead clinician. The Refreshed Framework for Maternity Care calls for the lead professional to be identified for all women by implementing national pathways and models of care. Symon et al. [4], however, called into question the specificity of the risk assessment tools used to allocate care pathways for women in view of Cheyne et al.’s [9] finding that 50% of women were at a high risk of obstetric complications at the end of pregnancy. Symon et al. [4] made the assumption that half of the Freestanding Maternity Unit (FMU) cohort in their study did not give birth at the FMU as they were ineligible due to obstetric risk factors, however maternal choice in the absence of complications may also have been influential. In this CMU study, only 11.9% of the women changed their care pathway from midwife-led to obstetrician-led as antenatal obstetric complications or “risk factors” developed which may reveal that the detail in the data allows the differences between women’s clinical indications and individual preferences about place of birth to become clearer. This would suggest that the risk assessment at booking for the CMU cohort of women allowed an accurate allocation of care pathway but some women chose, despite their lack of clinical complications, to give birth at the Obstetric Unit.

An interesting finding, in view of the low number (3%) of annual births occurring at CMUs [30], was that almost all women who were following a midwife-led pathway at 36 weeks named their CMU as their preferred birthplace . Whilst the reasons for these decisions cannot be explored in this paper, the choices women made through labour and birth may add some insights into why most women intended to give birth at CMUs. The pain management options available at the CMUs differed in that only the Cherrytrees team were able to offer women access to birthing pools. Intramuscular morphine was used by over twice the number of women at Seaview than those at Cherrytrees, and over twice the number of babies (3.6%) born at Seaview required resuscitation at birth compared to (1.2%) those born at Cherrytrees . Whilst significance cannot be drawn from these findings in this small descriptive study, the Cochrane systematic review of immersion in water in labour and birth [37] noted a decreased use of opiates (of which Morphine is one) by women labouring in water, but no differences in neonatal unit admissions or morbidity at birth compared to those who did not use water in labour. Of note too was that almost three quarters (72%) of all the births at Cherrytrees were waterbirths. This exceeded the rate derived from a national study of just over half (58.3%) of women who used a birthpool in labour [38]. Symon et al. [4] noted the ‘eclectic’ nature of individual freestanding maternity units (or CMUs), where explanations for anomalies in the process and outcomes of care delivery in discrete local contexts require further exploration.

### Limitations

This study has some limitations that need to be taken into account when determining its value in informing future practice and service development and in making a contribution to the evidence base. Of the five CMUs in Scotland which reported over 150 births annually, the CMUs included in the study were the only two which fulfilled the criteria of being rural and providing a midwife led model of care.

The data were collected retrospectively from case notes where it was originally recorded for a different purpose, therefore only secondary analysis of the data collected could be achieved. Contemporaneous and comprehensive clinical record keeping in the case notes could not be guaranteed. An alternative could be using electronic data collection methods. In a comparison between antenatal clinical data recorded in hand held maternity records and electronic records, however, neither achieved complete records when reviewed for best practice outcomes. [39].

The statistical data collected allowed a description of the frequency and percentage of the characteristics of the women who accessed maternity care at the CMUs, the process, outcomes and appropriateness of the care provided. More sophisticated statistical analysis was not required to achieve the research objectives, nor was it possible due to the low frequencies within certain variables, such as complications and morbidities, so inferences and associations between processes and outcomes cannot be made. A proportion (*n* = 45, 11.9% for Seaview and *n* = 26, 8.6% for Cherrytrees) of full records were missing and it cannot be assumed that these records would not have influenced the descriptive results achieved.

### Strengths

The strength of this research is the use of comprehensive data from of all women who accessed care at the CMUs. Many studies of maternity services excluded women with pregnancy complications, e.g. [36, 40, 41], and so this group of women are underrepresented against the rising trend of women with more complex needs accessing maternity care [15, 42].

This study adds original, contemporary evidence to the data required to inform policy decisions on maternity services. The original evidence presented in this paper suggests that the CMU model helped address health inequalities in pregnancy [12] by providing a locally based, but medically inclusive maternity care for most women in the local community with a mixed risk of obstetric complications.

## Conclusion

The CMUs provided a consistently high standard of safe and effective local inclusive maternity services when measured against national standards and international evidence. These services were accessible within the women’s local community and made a demonstrable contribution to current government policy of quality healthcare [11] and the integration of health and social care [43].

Future research recommendations would include a multi-centre exploration of maternity services provided at all CMUs in Scotland to build on the evidence presented in this paper about the quality of care provided through the CMU model to most women [44].

## Abbreviations

CMU: Community Maternity Unit
HEAT: Health improvement, Efficiency and governance, Access to services and Treatment appropriate
HQS: Healthcare Quality Strategy
NHS: National Health Service
